# Tactile perception of randomness: Effect of varying stimulus size and participants age

**DOI:** 10.1177/20416695231214954

**Published:** 2023-11-27

**Authors:** Mounia Ziat, Kayla Pacic, Ian Buentello, Joseph Varney, Fiona N. Newell

**Affiliations:** 8243 Bentley University, USA; 5336 Northern Michigan University, USA; 8809 Trinity College Dublin, Ireland

**Keywords:** randomness, tactile perception, aging, stimuli size

## Abstract

We investigated participants’ ability to differentiate between random and organized two-dimensional tactile tiles with embossed dots and examined how this ability varies with size and participant age. Four experiments were conducted to evaluate the effect of these variations on participants’ capacity to utilize touch in identifying which of two stimuli exhibited greater randomness. Participants were instructed to explore embossed tiles using both hands. The tiles had varying levels of randomness from organized to random sets. In Experiments 1, 2, and 4, the sets were of equal size, while in Experiment 3, they differed in size. Results revealed a significant difference between the random and organized sets, with random stimuli being more easily discernible. These findings suggest that touch can be utilized to discern random patterns on tactile maps or displays. However, older participants encountered difficulties making this distinction, indicating similarities between vision and touch in perceiving randomness.

Our perceptions are fundamental in shaping our understanding of and interactions with the world. One critical aspect facilitating this process is perceptual organization extensively studied across various modalities (Bregman, 1994; Lowe, 2012; McAdams & Bigand, 1993; Yoshioka et al., 2007). Perceptual organization, rooted in Gestalt principles, forms a strong foundation for comprehending how we organize and perceive multiple stimuli, whether grouped together or separately. When exploring a given space or perceptual field (Thomas, 2013), exploratory strategies (Klatzky et al., 1989) help us comprehend how individual stimuli are combined to form a holistic representation.

Among Gestalt principles, the concept of perceptual similarity is particularly essential as it forms the basis for comparing sets of features, even in touch (Lederman & Taylor, 1972). Similarity refers to the principle that stimuli sharing similar attributes, such as shape, size, color, texture, and orientation, are perceptually grouped together. This principle holds significant relevance in texture perception, encompassing visual and tactile domains.

In touch, Chang et al. (Chang et al., 2007) conducted a study demonstrating that participants employ tactile grouping strategies similar to vision by using texture to group by similarity and spatial positioning to group elements by proximity (elements close together are grouped together). However, the textures used in these investigations focused on organized, repeatable geometric patterns. Surfaces encountered in our ecological environment often present random patterns that may not adhere to the same organizational features or geometric metrics (see Figure 1). In fact, human texture perception encompasses three essential dimensions: randomness, periodicity, and directionality (Liu & Picard, 1994). Randomness represents one of these dimensions and is frequently observed in textures found in our everyday surroundings, contributing to the rich variety of perceptual experiences.

**Figure 1.**
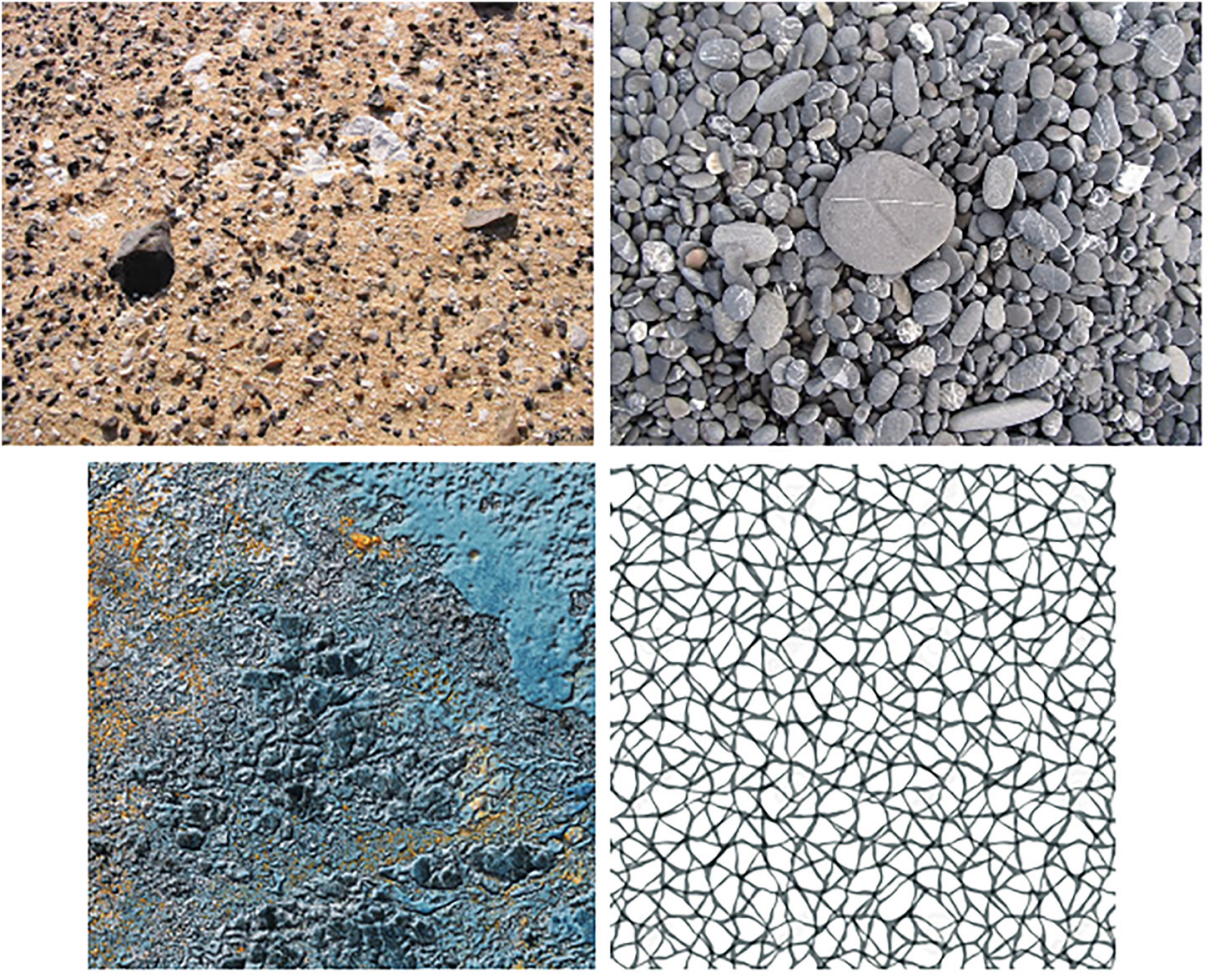
Examples of ecological random textures: top: left: sands and rocks and right: rocks; bottom: left: random rust and right: random grid.

The human perception of randomness is closely tied to the notion of algorithm complexity (Gauvrit et al., 2014), with implications for pattern recognition and computational models used for spatial pattern identification and classification (Fujii et al., 2003). This understanding finds practical applications in diverse fields, such as ecology, for studying sets of trees or animal populations, and astronomy for the analysis of astronomical data (Ginsburg, 1997). Despite lacking biological sensors for precise distance measurement, human exhibit remarkable estimation skills in length magnitude estimation Stevens (1962). Additionally, their ability to group objects into clusters based on principles like similarity and proximity contributes in estimating the distance between two dots or clustering proximate dots (Guberman, 2015).

In this article, we investigated humans’ ability to distinguish the concept of randomness from the concept of organization by asking participants to compare tactile stimuli (convex dots) with different levels of randomness (Experiments 1, 2, and 3). While there is a visual and complexity difference between the ecological textures (Figure 1) and our experimental stimuli, the latter was purposefully designed to enable a controlled and focused study of randomness perception. In vision, while previous studies have explored randomly positioned stimuli (Wolfe, 1994), the task becomes more challenging when dealing with complex textures. When participants were asked to compare the regularity of botanical structures in a two-forced choice task, they encountered difficulties in identifying organizational structures within the images (Fujii et al., 2003). In touch, most research on two-dimensional (2D) exploration has predominantly focused on features such as repetition, direction, compliance, texture segregation, and tactile acuity (Goodwin et al., 1989; Katz et al., 1925; Lederman & Taylor, 1972; Legge et al., 2008; Sathian et al., 1989; Whitaker et al., 2008).

However, not all studies on tactile texture perception have used simple geometric patterns. In a study exploring the perception of surface statistics, Kuroki et al. (Kuroki et al., 2021) found that while human perception in other modalities, like vision, can utilize both lower- and higher-order image statistics, the tactile system primarily relies on lower-order statistics for texture discrimination, even when presented with surfaces transcribed from natural scenes. In our current work, while we maintain consistent dot properties, the spatial arrangement and perceived randomness of these dots vary. Specifically, our stimuli can be considered as first- or second-order statistics. Although the individual dot properties (size and shape) remain consistent across stimuli, the overall spatial mean might shift depending on their random placement.

Additionally, we examined whether the ability to perceive randomness changes with age (Experiment 4). This latter was partly inspired by Yamada’s research, which has shown that individuals over 60 had difficulty discriminating random visual stimuli compared to those under 40 years old and that female participants exhibited a better ability to estimate randomness than their male counterparts (Yamada, 2015).

As we age, our visual and auditory capabilities may decline (Bell et al., 1972; Bennett et al., 2007; Gilmore et al., 1992; Gittings & Fozard, 1986; Goh et al., 2007; Roth, 2015), while this decline in our sense of touch remains comparatively resilient. However, it is worth noting that individuals older than 65 may experience a reduction or alteration in tactile sensations and acuity (Manning & Tremblay, 2006; Stevens, 1992; Woodward, 1993; Wickremaratchi & Llewelyn, 2006). Due to the natural decrease in tactile sensitivity that can occur with age, some individuals have reported difficulty in discriminating certain textures (Norman et al., 2016; Verrillo et al., 1998). Fortunately, skin hydration can partially restore this sensitivity (Lévêque et al., 2000). Several factors contribute to this loss of sensitivity, including a decline in skin elasticity (Amaied et al., 2015) and a decrease in selective attention, which hinders our ability to focus on multiple touch stimuli (Valeriani et al., 2003). Furthermore, age and gender can also influence factors such as mechanoreceptor density (Cauna & Ross, 1960), the central nervous system (Nusbaum, 1999), and the nature of touch (static vs. dynamic) (Abdouni et al., 2018).

In the following sections, we present a series of experiments centered on understanding the perception of randomness in tactile patterns. Experiment 1 introduces our investigation by examining individuals’ abilities to discriminate tactile patterns based on their randomness levels. Experiment 2 looks into the impact of stimulus size on performance, exploring how changes in the spatial area and, therefore, dot proximity influence perception. Experiment 3 further investigates the intricacies of tactile randomness perception by exploring areas of different sizes, while Experiment 4 assesses this perception across multiple age groups.

For Experiment 1, we expected participants to differentiate between tiles with perfectly aligned dots and those with randomly scattered dots. In Experiment 2, reducing the surface of the tactile stimuli while maintaining the size of the dots was hypothesized to result in a decline in participants’ performance in differentiating between random and organized patterns. In Experiment 3, we anticipated that the size congruency of tactile tiles would affect participants’ ability to distinguish between levels of randomness. Finally, in Experiment 4, we expected age to influence the perception of randomness in tactile tiles significantly.

Before the experiments, we conducted a power analysis to determine the optimal number of participants. Using G*Power, we aimed to detect a small effect size (*f* = 0.30) in a repeated measures analysis of variance (ANOVA), with a significance level ( α ) of 0.05 and a desired power of 0.80. The recommended total sample size was determined to be 10. Our smallest sample size, 12 participants in Experiment 3, was sufficiently powered to detect a small effect. Detecting subtle effects requires a larger sample size for adequate power. However, the number of measurements (10 repetitions) helps reduce the sample size.

## Experiment 1

### Method

#### Participants

Twenty-seven students (14 F, 13 M) from Northern Michigan University (NMU), aged 18 to 25 (mean = 22.45, *SD* = 1.52), were recruited for this study. Before the experiment, the participants provided informed consent and received class credit for their participation. The experimental procedures followed the guidelines of the Declaration of Helsinki, and the study protocol received approval from the Institutional Review Board (IRB) at Northern Michigan University.

#### Stimuli

Our stimuli comprised 2D squared tiles 3 × 3 cm, each containing 49 embossed dots. The dots were either perfectly aligned or randomly scattered on a grid. In spatial randomness modeling, distance analysis and quadrat analysis are commonly employed techniques to study the arrangement of events in a given area (Diggle, 2013). These approaches draw inspiration from human psychophysical experiments that use the principles of perceptual organization and Gestalt psychology. Distance analysis, for example, examines the distances between points to assess whether a set of dots exhibits organization or randomness. In contrast, quadrat analysis focuses on the number of dots that fall within a specific shape, determining whether they follow a random pattern or a predefined arrangement. To determine the placement of the 49 embossed dots, we used a continuous uniform probability density function with mean μ and range *r* in the *x* and *y* dimensions. This approach employed by Yamada in 2015 was used to generate random visual stimuli (Yamada, 2015).

(1) $$f(X)=\left\{ \begin{array}{cc} \frac{1}{r} & \mathrm{for}\;(\mu -\frac{r}{2})<X<(\mu +\frac{r}{2})\\ 0 & \mathrm{otherwise} \end{array}\right.$$

The level of randomness in the pattern increased as the value of *r* became larger. To produce the stimuli patterns, we utilized Matlab to create the desired visual images. These images were then transcribed onto cast acrylic tiles using a 2D laser engraver. During the engraving process, specific layers of the acrylic were selectively removed, leaving behind convex dots that stood out from the surface. Each of these convex dots had a diameter of ∼ 1.5 mm and a height of 0.43 mm. The resulting tactile tiles consisted of one standard stimulus (R10 with r=10 pixels) with an intermediate level of randomness and eight comparison stimuli. On a perfectly aligned grid, the dots were spaced 3 mm apart; this spacing was subject to random variation as the value of *r* increased.

**Figure 2.**
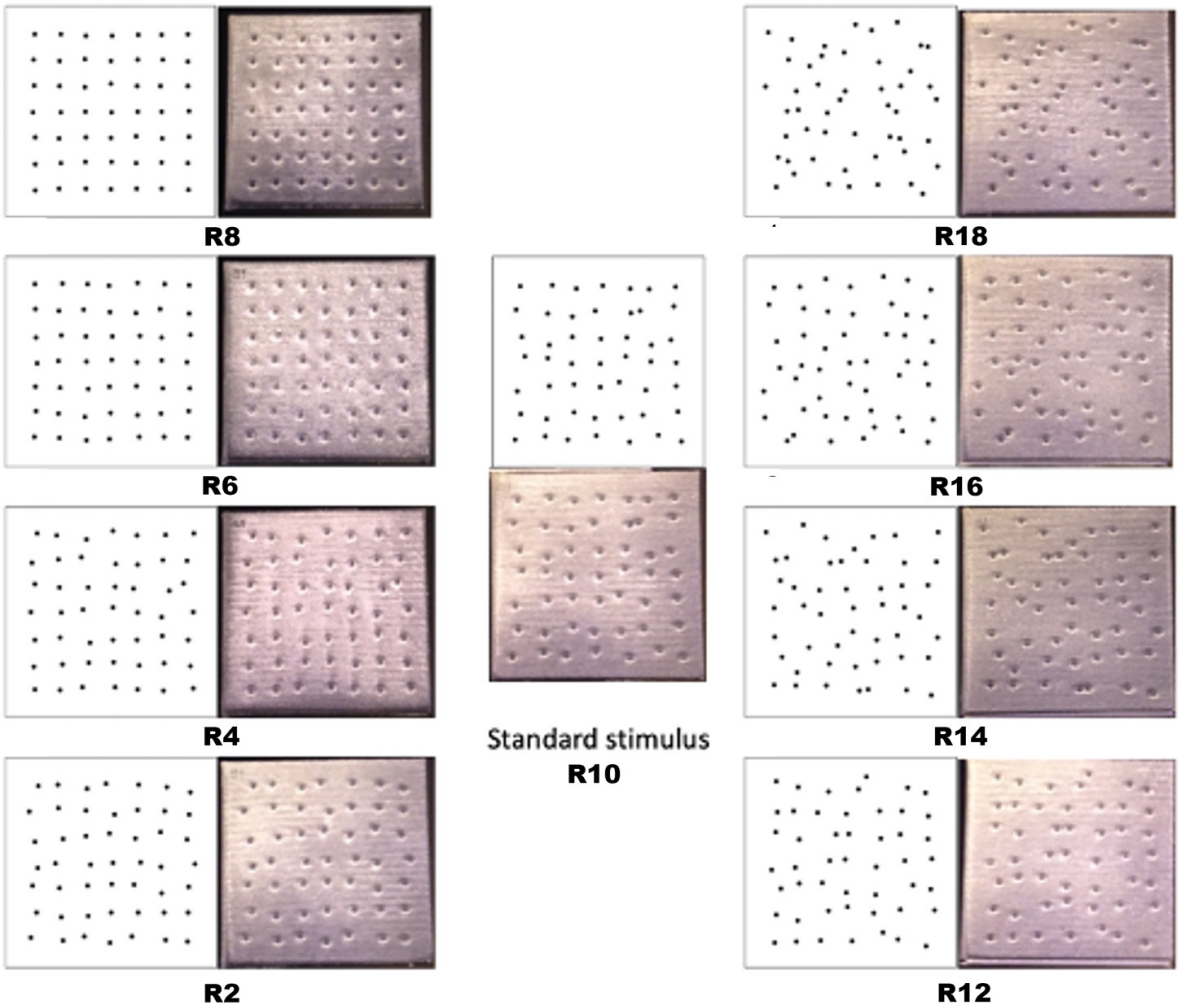
The two sets of comparison stimuli: organized (R2–R8) and random (R12–R18), along with the standard stimulus (R10). The visual models were used to print the nine stimuli into cast acrylic tiles with a thickness of 0.6 cm

Four of the comparison stimuli were classified as “Organized” because they were systematically less random than the standard stimulus. These stimuli ranged from R8 (8 pixels), R6 (6 pixels), R4 (4 pixels), to R2 (2 pixels), with 2-pixel decrements. The remaining four comparison stimuli were categorized as “Random” as they were systematically more random relative to the standard stimulus. These stimuli ranged from R12 (12 pixels), R14 (14 pixels), R16 (16 pixels), to R18 (18 pixels), with 2-pixel increments. Figure 2 illustrates the visual patterns and their corresponding printed tiles.

#### Procedure

After signing the consent form, participants were directed to sit in a comfortable chair in front of a table, where stimuli were presented out of their sight. All participants engaged in a 2AFC (two-alternative forced choice) discrimination task that involved exploring 2D tactile stimuli using their index fingers. Stimuli were explored using both hands simultaneously, restricted explicitly to the index fingers. The stimuli consisted of two square tiles, each containing 49 raised dots. Eight comparison stimuli, comprising four organized patterns and four random patterns, were compared against a standard stimulus with an intermediate level of randomness.

Before commencing the main task, participants were introduced to the concept of randomness versus organized tactile patterns through a multi-modal approach. Initially, visual stimuli were presented on paper to illustrate the difference: some dots were depicted as perfectly aligned on a grid, while others were shown as deviating from this alignment (refer to the visual illustration in Figure 2. Participants were then encouraged to physically explore the tactile tiles, allowing them to bridge the visual understanding with the tactile experience. To ensure comprehension and comfort with the task, each participant undertook five practice trials while blindfolded, during which they received feedback on their responses.

The task encompassed a total of 40 trials, which were presented twice in two separate blocks with a 5-minute break in-between. In the first block, the standard stimulus were presented to one hand (e.g., left), while the comparison stimuli were presented to the other hand. In the second block, the standard stimulus was presented to the opposite hand (e.g., right) compared to the first block. The order of both the tiles and handedness was randomized across participants. Participants were blindfolded throughout the task and were asked to determine which stimulus appeared to have a more randomized pattern and respond by indicating whether it was on the left or right side. The exploration time for each trial lasted between 5 and 10 s.

#### Results and Discussion

A one-way repeated measures ANOVA was conducted to examine the effects of the main factor *Level* of randomness, which ranged from R2 to R18 in 2-pixel increments. The assumption of sphericity was not violated ( p>.05 ), and the analysis of variance revealed a significant main effect of the *Level* of randomness [*F*(7,182) = 17.40, p<.001 , $\eta_{p}^{2}\;=\;0.40$ ].

**Figure 3.**
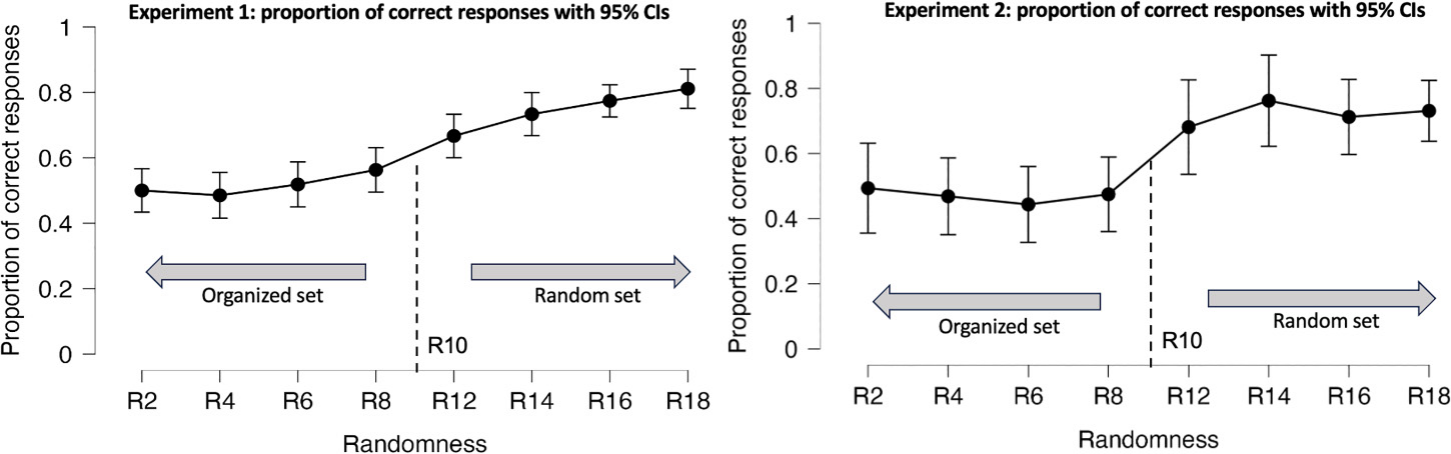
Proportion of correct answers by randomness levels for Experiment 1 (left) and Experiment 2 (right), with error bars representing 95% confidence interval values. Organized and random sets are displayed in reference to the standard stimulus, R10.

Post-hoc analysis using the Bonferroni correction revealed that each stimulus from the Organized set (R2–R8) significantly differed from each stimulus of the Random set (R12–R18) ( p<.05 ) (Figure 3). Additionally, R12 was significantly different from R18. The findings suggest that participants could classify the random set relative to the standard stimulus but struggled to differentiate the standard stimulus from the organized set. This outcome supports the notion that perceiving a significant disruption in a texture is easier than detecting a subtle disturbance in an already organized texture.

## Experiment 2

### Method

In this second experiment, we aimed to examine the impact of stimulus size on performance. Similar to acuity tests, our objective was to investigate how reducing the surface of a stimulus while keeping its properties unchanged (i.e., size of the dots remain the same), would affect performance. We hypothesized that decreasing the distance between dots in the pattern would lead to a decline in performance. Additionally, we anticipated that the smaller spatial area resulting from the reduced size would reduce the range of active exploration.

#### Participants

Sixteen NMU students (9 F, 7 M) between 19 and 25 years old (mean: 22.1, *SD* = 2.9) took part in the study and received class credit for their participation. After signing the consent form, participants were seated on a chair and were informed they could withdraw at any time during the experiment. The study was approved by NMU IRB.

#### Stimuli

For Experiment 2, the stimuli consist of one standard stimulus R10 (intermediate level of randomness) and eight comparison stimuli, as described in Experiment 1. The size of the tiles was reduced to 2×2 cm to assess the impact of closer dots on the perception of randomness. While the diameter and height of the convex dots remained the same, the inter-dot distance was reduced to 2 mm. The procedure followed the same protocol as in Experiment 1.

#### Results and Discussion

The analysis of variance with a Greenhouse-Geisser correction revealed a significant effect of the factor *Level* of randomness, *F*(7,105) = 5.57, p<.05 , $\eta_{p}^{2}=0.30$ . Post-hoc analysis revealed significant differences between the random and organized sets across all levels. However, unlike in Experiment 1, the randomness levels R12 and R18 did not show significant differences, suggesting a lower sensitivity for smaller area sizes. Figure 3 shows that the performance dropped down for higher levels of randomness.

Participants’ comments suggested they opt for a more global exploration as the area size becomes smaller ( 2×2 cm). The increased space in the 3×3 cm tiles allowed participants to actively explore using their index fingers and identify specific features that aid identification. Conversely, with a 2×2 cm tile, the reduced space may hinder their ability to do so. Comparing the results of Experiment 1 to Experiment 2, independent *t*-tests revealed no significant differences for all eight levels of randomness.

## Experiment 3

### Method

In Experiment 3, our objective was to examine the impact of size congruence on the perception of randomness. Participants were presented with stimuli of two different sizes and were asked to perform a bimanual discrimination task using their index fingers. This experiment aimed to shed light on how size congruency influences the ability to discriminate between stimuli that vary in levels of randomness. The findings could have practical implications in fields such as tactile design, where understanding the influence of size on perception can inform the design of more effective tactile interfaces.

#### Participants

Twelve NMU students (9 F, 7 M) between 20 and 23 years old (mean: 21.4, *SD* = 1.3) participated in the study and received class credit for their time. After signing the consent form, participants were seated on a chair and were informed they could withdraw at any time during the experiment. NMU IRB approved the study.

#### Stimuli

The stimuli set included a standard stimulus R10 (intermediate level of randomness) measuring 3×3 cm and eight comparison stimuli with a tile size of 2×2 cm. The procedure followed a similar format as the previous experiments, consisting of a total of 80 trials.

#### Results and Discussion

**Figure 4.**
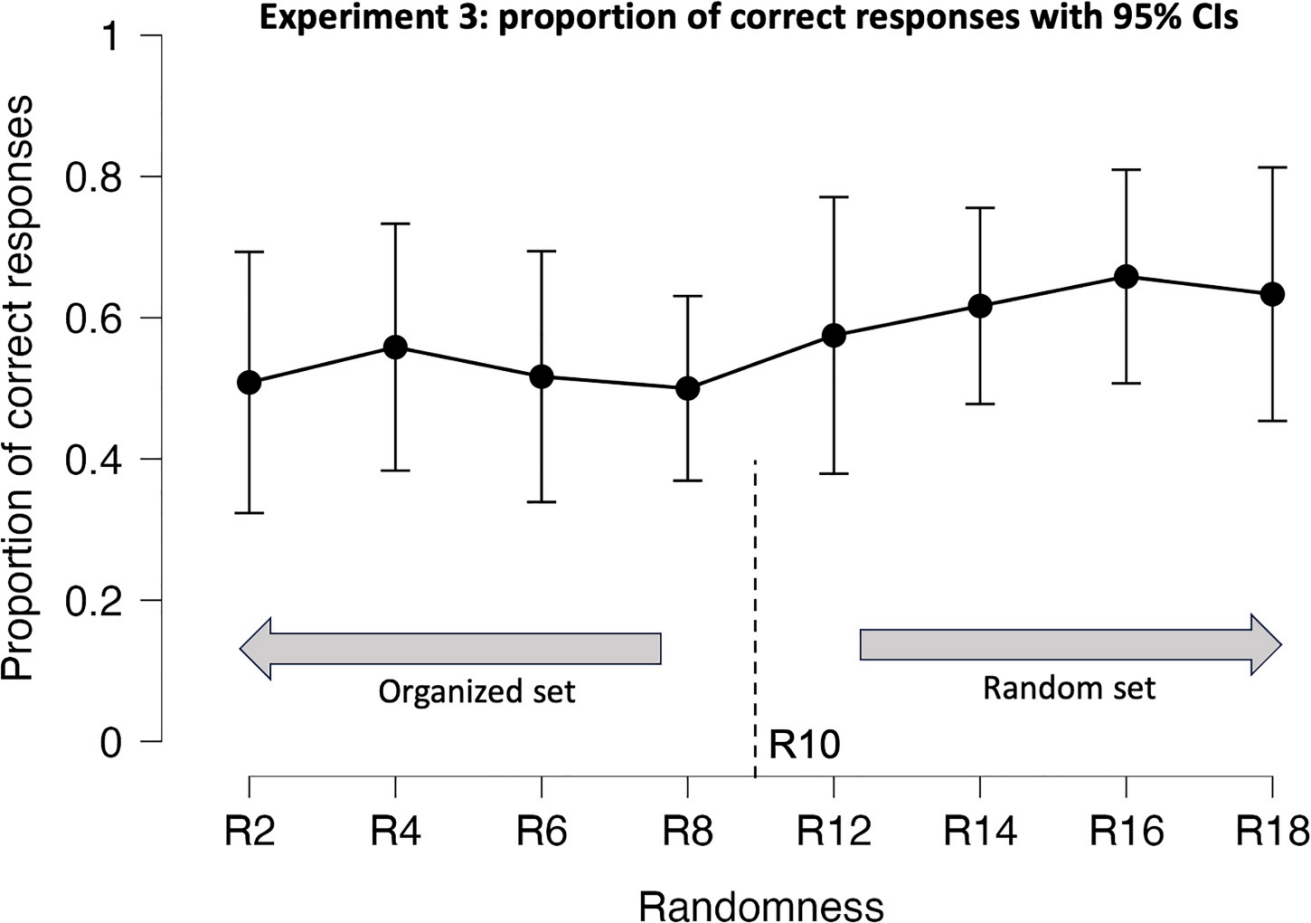
Proportion of correct answers by randomness levels for Experiment 3, with error bars representing 95% confidence interval values. Organized and random sets are displayed relative to the standard stimulus R10.

The results of Experiment 3 revealed a negative impact on participants’ ability to accurately perceive randomness in tactile stimuli when presented in different sizes (Figure 4). While the analysis of variance did not identify significant effects for the main factor, level of randomness ( p>.05 ), participants’ overall performance closely aligned with chance levels. This observation was further supported by one-sample *t*-tests: aside from the performance for R16 [t (11) = 2.56, p<.05 , Cohen’s *d* = 0.74], the responses across other levels were not statistically different from a 50% chance level ( p>.05 ). Participants reported experiencing difficulties when exploring surfaces of varying sizes, which hindered their ability to identify the arrangement of the dots accurately.

Previous studies have indicated that altering the size of objects can adversely affect participants’ performance in both 2D (Reed et al., 1990) and 3D (Craddock & Lawson, 2009) object exploration, which may explain the observed results. Independent *t*-tests comparing Experiment 3 to Experiment 1, across all eight levels, revealed a significant decline in performance for nearly all random levels: R14 [*t*(37) = 1.7, p<.05 , Cohen’s *d* = 0.45], R16 [*t*(37) = 1.99, p<.05 , Cohen’s *d* = 0.59], and R18 [*t*(37) = 2.42, p<.05 , Cohen’s *d* = 0.69]; with no significant difference for the organized levels. Additionally, no significant difference was found between Experiment 2 and Experiment 3. These findings suggest that further research is necessary to fully comprehend the impact of size on tactile 2D bimanual exploration.

## Experiment 4

### Method

#### Participants

The final experiment aimed to investigate the relationship between age and the perception of randomness. To conduct this investigation, a total of 100 participants were recruited. The participants’ demographic details, based on age categories and gender, is summarized in Table 1. The experiment was conducted in collaboration with a medical doctor at their practice in Iron Mountains, MI. This approach allowed for a broader representation of age groups beyond the typical college student population. A sweepstake was held to encourage participation, offering a chance to win an iPad mini tablet. The winner was notified via email and provided with instructions on how to collect the prize. The experimental procedure was approved by the IRB of NMU.

**Table 1.** Participants’ distribution per age and gender.

Category	Number	Mean age	Gender
Age 5–10	6	8.5	1 F, 5 M
Age 11–20	16	16.88	12 F, 4 M
Age 21–30	27	24.85	20 F, 7 M
Age 31–40	25	35.76	16 F, 9 M
Age 41–50	12	47.33	7 F, 5 M
Age 51–60	14	55.07	9 F, 5 M

Abbreviations: F = female; M = male.

#### Stimuli and Procedure

The stimuli used in the experiment were identical to those used in Experiment 1. They consisted of 2D square tiles measuring 3 × 3 cm, each containing 49 dots. The procedure followed a similar format to the previous experiments, except that we did not provide specific instructions for exploring the tiles, nor did we impose a time limit to prevent bias among age groups.

Similarly, participants were tasked with determining which of the two presented tiles had the most randomized pattern. To ensure comprehension among younger children, the experimenter provided a simplified explanation of the concept of randomness by showing the tiles while asking the children to touch them simultaneously. Children’s understanding of perceptual organization typically develops around the age of two. Prior research has demonstrated that by ages 2 and 5, children exhibit an adult-like understanding of visual acuity tasks, such as a grating or vernier acuity, respectively (Zanker et al., 1992). Additionally, concepts such as visual segmentation and texture are generally comprehended by age 2 (Atkinson & Braddick, 1992; Kovács, 2000; Rieth & Sireteanu, 1994; Sireteanu & Rieth, 1992). In our experiment, the youngest participant was six years old.

### Results and Discussion

A mixed-design ANOVA was conducted on the data from Experiment 4 to investigate the effects of age and gender on the perception of randomness. The within-subjects factor was the Level of randomness, while Age and Gender were the between-subjects factors. Both Mauchly’s test for sphericity and Levene’s test for homogeneity of variances confirmed that the assumptions were met ( p>.05 for both tests). The ANOVA results showed a significant main effect for randomness [*F*(7,616) = 9.23, p<.001 ]. Notably, an interaction effect between randomness and age was observed [*F*(7,616) = 2.77, p<.05 ]. Exploring this interaction via pairwise comparisons revealed that all randomness levels within the organized set were significantly different from those in the random set across age groups ( p<.001 ). The only exception was the 51–60 years old group, which demonstrated no discernable performance difference between the two sets. The analysis did not find gender significantly influencing the perception of randomness, and no further interaction effects emerged.

**Figure 5.**
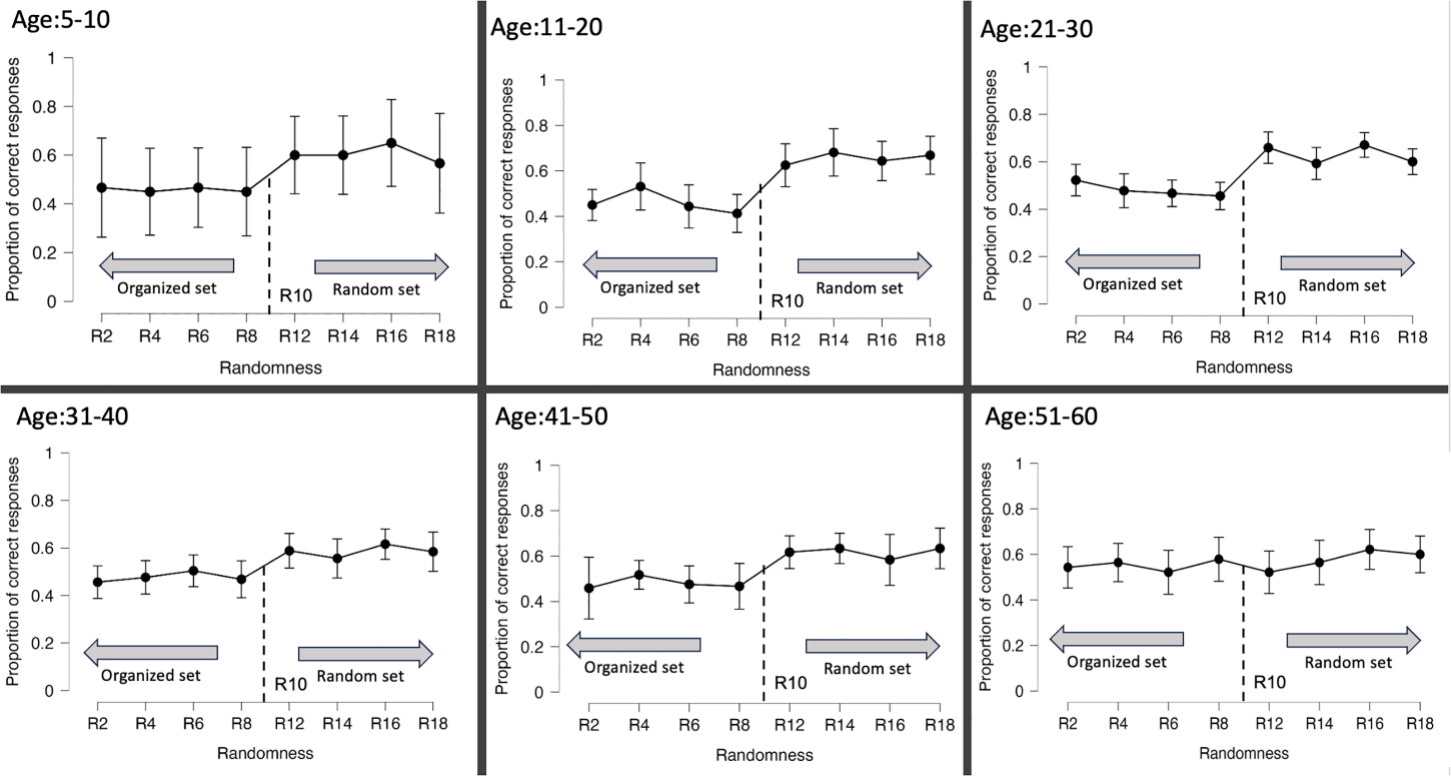
Proportion of correct answers for all age categories and randomness levels, with error bars representing 95% confidence interval values. The standard stimulus (R10) line divides the graphs into two sets: organized and random sets.

These findings suggest that the ability to perceive randomness in 2D tactile stimuli remains consistent across different age groups. Participants exhibited higher accuracy in identifying the more random stimuli than the organized stimuli, except for the 51–60 years old group, who showed comparable performance for both sets. The results are further illustrated in Figure 5. The absence of differentiation between the two sets in the older participants can be attributed to a decline in tactile acuity that affects performances in many haptic tasks (Manning & Tremblay, 2006; Norman et al., 2016). Regrettably, we could not measure participants’ acuity due to time constraints during routine doctor visits.

## General discussion

Our findings indicate that performance in the Random category significantly influenced the task outcomes. Participants were more successful in identifying stimuli as more random when compared to a standard stimulus rather than identifying stimuli as less random. This trend was consistent across all age groups, with one exception: participants in the 51–60 age range exhibited a weakened distinction. Notably, the older participants did not perceive random patterns as significantly different from organized patterns, suggesting uncertainty when comparing both sets. This observation aligns with Yamada’s study on visual randomness in relation to age (Yamada, 2015). Conducting further investigations with participants over 60 might unveil a decline in their tactile ability to distinguish random patterns from those observed visually.

Additionally, the size of the tiles slightly affected the perception of randomness, making it more challenging to differentiate between different levels of randomness. This finding aligns with previous research demonstrating how reducing the size of a texture makes it more difficult to distinguish between similar or dissimilar textures placed side by side (Chimata & Schwartz, 2018). During the debriefing sessions, participants consistently highlighted the influence of tile size on their exploratory procedures. For smaller tiles, the limited exploration space, combined with the size of the fingertip, naturally encouraged a more global exploration approach. Given the constrained surface area, participants felt they could grasp the overall texture without the need to focus on specific regions. In contrast, with larger tiles, participants had more freedom to navigate and often chose to concentrate on particular areas, allowing for a more localized and detailed exploration. As the 2D tactile exploration was limited to index fingers, participants relied on active touch to examine the tiles. Previous studies have shown a preference for one-finger exploration (Craig, 1985), with no observed advantages for using two fingers over one finger when engaging in 2D exploration (Jansson & Monaci, 2003; Loomis et al., 1991).

One of the most relevant studies to this research is the work conducted by Ballesteros et al. on symmetry tasks in the haptic modality (Ballesteros et al., 1997, 1998). Although their study did not explicitly address randomness, the authors discovered that it was easier to discriminate asymmetrical shapes, such as raised dots or lines, compared to symmetrical ones. They attributed this phenomenon to several factors, including a lower cognitive load involved in scanning random stimuli as opposed to organized or symmetrical stimuli. Furthermore, discriminating organized or symmetrical stimuli during bimanual exploration requires a spatial reference, which is absent in touch perception without prior training. As suggested by Millar (Millar, 1978), when participants explore random raised dots, they tend to code texture differences rather than spatial organization since there are no spatial referent, to begin with. Consequently, comparing organized stimuli becomes more challenging. Supporting these observations, a related study (Pappas et al., 2009) found that random patterns were described as “interesting” and stood out significantly from the symmetric, more regular patterns.

An area for future research could involve combining sets of similar or more complex stimuli with visual cues. While Yamada’s study demonstrated that random dots could be easily discriminated visually, it is known that humans struggle to identify regular patterns in more complex stimuli or high-order statistics, as observed by Fujii et al. (Fujii et al., 2003) and in Kuroki et al.’s research (Kuroki et al., 2021), respectively. This difficulty may be attributed to the nature of the task, which involves discriminating regularity rather than randomness. The concept of randomness is generally easier to comprehend than that of regularity, as regularity entails following specific rules that may be predetermined by the experimenter or chosen by participants. In vision, discriminating misaligned or distinguished features is less effortless. This process is similar in the haptic modality when participants are presented with a larger spatial area. We found that, except for Experiment 1, there were no perceived differences in the levels of randomness, which may be influenced by the size of the tiles ( 3×3 cm) or larger. It would be interesting to explore additional tile sizes in future studies.

Finally, understanding how organisms process randomness in the real world is crucial for explaining why random stimuli are more easily perceived. In visual perception, such as motion, randomness plays a significant role in ensuring survival (Gauvrit et al., 2014). It enables animals to evade predators or capture prey (Geisler, 2008) and facilitates tracking a target or reaching a destination. While our study did not simulate threatening conditions for participants, these behaviors are deeply ingrained in our ecological heritage. They can unconsciously shape our learning and perception, as with latent learning (Seward, 1949).
